# The Effect of Preoperative Imaging on the Negative Appendicectomy Rate

**DOI:** 10.7759/cureus.41809

**Published:** 2023-07-13

**Authors:** Jamal Dirie, Humza Suleman, Hussain Karimjee

**Affiliations:** 1 General Surgery, Royal Surrey NHS Foundation Trust, Guildford, GBR

**Keywords:** preoperative imaging, negative appendicectomy rate, appendectomy, negative appendectomy rate, appendicitis

## Abstract

Background

Appendicitis is one of the most common causes of acute abdominal pain and appendicectomy is one of the most frequently performed surgical procedures. The proliferation of radiological imaging has reduced the number of patients who have a normal appendix removed, i.e., a negative appendicectomy. We aimed to assess the effect of preoperative imaging on the negative appendicectomy rate (NAR).

Methodology

All emergency appendicectomies performed at a district general hospital in the United Kingdom over two separate one-year periods were retrospectively analysed using emergency theatre log books. The timeframes were chosen based on the introduction of a diagnostic pathway to reduce the number of appendicectomies performed on patients later found not to have appendicitis or alternative abnormality, i.e., a negative appendicectomy. This pathway involved a greater emphasis placed on preoperative imaging (CT or ultrasound) for patients with suspected appendicitis. The study excluded any patients who were found to have an alternative pathology during surgery. Information technology databases were used to collect data on patient demographics, date of surgery, histology, and any preoperative imaging that was performed. All histological findings showing acutely inflamed appendices and those positive for malignancy were categorised as positive, whereas all other findings were categorised as negative.

Results

During our initial data collection period (April 2018 to April 2019), we collected data on 207 patients who underwent an appendicectomy. The NAR was 17%. During our subsequent data collection period (August 2020 to August 2021), we collected data on 184 patients. The NAR was 16%. In our adult population, the NAR decreased from 13% to 9%.

Discussion

At first glance, the NAR does not seem to have improved. On closer look, all patients over the age of 21 years in our re-audit underwent pr-operative CT, and there was a reduction in the NAR in these patients. The issue arises with younger patients, in whom justifying the radiation associated with a CT scan may be difficult. Although ultrasound does not carry the same radiation risk, previous audits at our trust have that shown its sensitivity and specificity for appendicitis is approximately 60%. We may have to explore alternative imaging modalities such as MRI in the paediatric population or accept the higher NAR.

## Introduction

Acute appendicitis is a common surgical emergency worldwide, with an estimated lifetime risk of 7-8% [1]. Appendicectomy, the surgical removal of the appendix, is the standard treatment for this condition [2]. However, a significant proportion of patients who undergo this surgery are found to have a normal appendix upon histopathological examination. This is known as a negative appendicectomy, and it can lead to unnecessary surgery, prolonged hospital stays, increased healthcare costs, and associated morbidity and mortality.

The incidence of negative appendicectomy varies widely, ranging from 8% to 45%, with considerable variation between different institutions and countries [3,4]. Several factors contribute to the likelihood of negative appendicectomy, including clinical and radiological features, as well as the experience and skill of the surgical team [5]. This paper aims to review the practice found in a district general hospital in the United Kingdom, and whether the introduction of a diagnostic pathway that emphasises increased use of imaging reduces the negative appendicectomy rate (NAR).

The incidence of acute appendicitis varies widely across different populations, with higher rates reported in Western countries and lower rates reported in Asian countries, although this difference is rapidly being bridged, possibly due to increasing industrialisation [6]. The incidence also varies according to age, with a peak incidence in the first and second decades of life [7].

The pathophysiology of acute appendicitis involves inflammation and obstruction of the appendix, leading to bacterial overgrowth and eventually perforation if left untreated. The exact cause of acute appendicitis is not well understood, but several factors have been implicated, including infection, diet, genetics, and environmental factors [8].

The diagnosis of acute appendicitis is primarily based on clinical presentation, including abdominal pain, fever, and leukocytosis. However, clinical presentation can be variable and atypical in certain populations, leading to diagnostic uncertainty [9]. Imaging studies, including ultrasound and CT, have been shown to improve the accuracy of diagnosis, particularly in atypical cases. However, the use of imaging studies can lead to a delay in care and has an associated radiation risk in the case of CT.

The management of acute appendicitis depends on the severity and duration of symptoms, as well as the risk of perforation. Appendicectomy is the standard treatment for this condition, but conservative management with antibiotics has been shown to be effective in selected cases [10]. The management of negative appendicectomy involves careful evaluation of the surgical technique, as well as consideration of alternative diagnoses and further investigations.

Several strategies have been proposed for reducing the incidence of negative appendicectomy, including the use of standardised diagnostic criteria, the adoption of imaging studies, the implementation of quality improvement initiatives, and the development of new diagnostic tools [11]. These strategies aim to improve the accuracy of diagnosis, reduce the risk of unnecessary surgery, and optimise the use of healthcare resources.

## Materials and methods

We performed a retrospective analysis of all emergency appendicectomies performed in a district general hospital in the United Kingdom over two separate one-year periods. We used emergency theatre logbooks to identify appropriate patients. Our inclusion criteria were all patients who had undergone an emergency appendicectomy. We excluded any patients who underwent an elective appendicectomy, and any patients who underwent an appendicectomy as part of more extensive surgery.

The timeframes were chosen based on the introduction of a diagnostic pathway that placed a greater emphasis on preoperative imaging (CT or ultrasound) to reduce the number of appendicectomies performed on patients later found not to have appendicitis or alternative abnormality. This pathway involved a greater emphasis placed on preoperative imaging for adult patients. The study excluded any patients who were found to have an alternative pathology during surgery, even if they had their appendix removed.

Electronic databases were used to collect data on patient demographics, date of surgery, and histology. We also identified any patients who underwent preoperative imaging. If patients had undergone preoperative imaging, we identified the modality, and whether the imaging study was positive for appendicitis or negative. Histological findings showing acutely inflamed appendices and those positive for malignancy were categorised as positive, whereas all other findings were categorised as negative. The histological categorisation was used to define a ‘negative’ vs ‘positive’ appendicectomy.

The data were organised into tables in Microsoft Excel. This was then used to calculate the NAR for patients undergoing imaging (CT or ultrasound) and patients who did not undergo imaging. The same software was used to analyse the data using descriptive statistics and represent the results in a visual format (bar charts or tables).

## Results

In our pre-intervention cohort, there were a total of 207 patients who underwent appendicectomy. The age range was 8 to 81 years. This is represented in Table 1. Only 32% of patients underwent preoperative CT, with a sensitivity of 96% and specificity of 90%. A further 31% of patients underwent preoperative ultrasound, with a sensitivity of 83% and specificity of 48%.

**Table 1:** Demographics in both pre and post-intervention groups.

	Pre-intervention	Post-intervention
Number of patients (total)	207	185
Age range (minimum–maximum)	8–81	9–80
Males (total)	96	85
Females (total)	111	100
Adults (>18)	149	152
Paediatrics (<19)	58	33

In our subsequent data collection period, there was a reduction in the total number of patients who underwent surgery, with only 185 patients who underwent appendicectomy. The age range was 9 to 80 years. In this cohort of patients, 65% of the patients underwent preoperative CT, with a sensitivity of 94% and specificity of 92%. A further 14 % underwent ultrasound, with a sensitivity of 74% and specificity of 53%.

**Figure 1:**
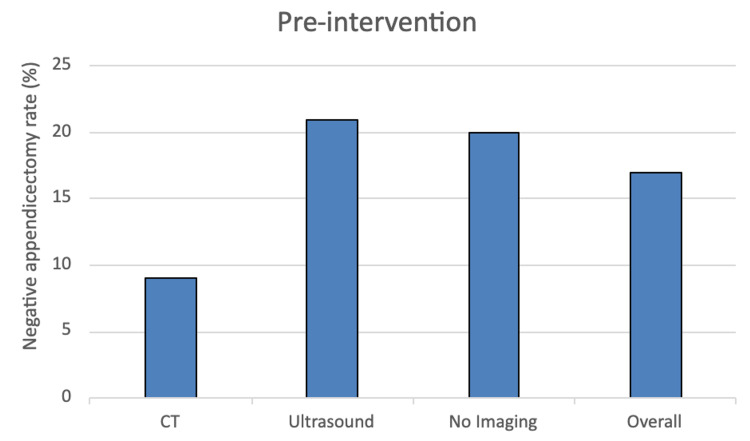
The variation in the negative appendicectomy rate before the introduction of our diagnostic pathway.

**Figure 2:**
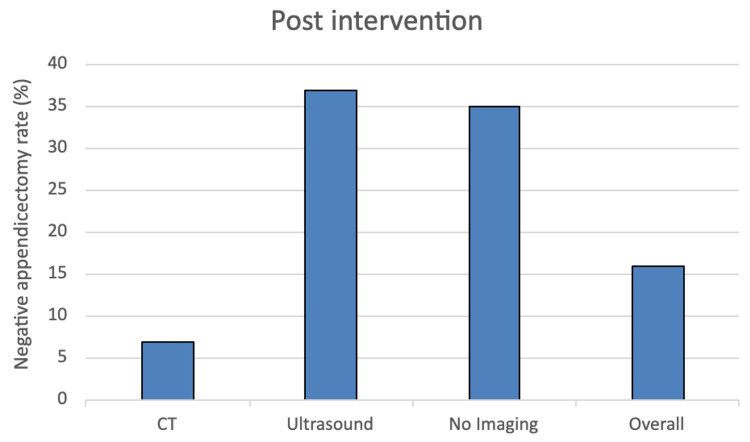
The variation in the negative appendicectomy rate after the introduction of our diagnostic pathway.

In the pre-intervention group, the NAR was 17%. In the post-intervention group, the NAR was 16%. In the adult population, the NAR decreased from 7% to 3%. In the paediatric population, the NAR increased from 35% to 41%. The different NARs based on CT, ultrasound or no imaging are represented in Figure 1 and Figure 2, for the pre and post-intervention groups, respectively.

## Discussion

Appendicitis is a very common cause of abdominal pain. Until recently, it was a clinical diagnosis, with patients being taken to the operating room based on signs, in conjunction with blood results. There are several scoring systems, including the Alvarado score, which attempt to combine the ‘classical’ signs of appendicitis with certain blood results, including white cell count and C-reactive protein, to predict the likelihood of appendicitis [12]. However, patients often do not present with classical symptoms, and therefore, the use of imaging has provided an excellent adjunct in the diagnosis of appendicitis.

The NAR does not appear to have improved significantly in our study. However, there are some key points to discuss regarding our results.

For instance, although the overall NAR did not improve, we saw an improvement in the NAR when looking at our adult cohort. This cohort of patients also saw an increase in the use of preoperative CT compared to our pre-intervention group. These two results combined are correlated with previous studies suggesting that increased use of preoperative imaging reduces the NAR [13]. Notwithstanding its effects on the NAR, the widespread availability of CT in the diagnosis of appendicitis has also had a positive effect on days in the hospital and cost when compared to a diagnostic laparoscopy for possible appendicitis. The number of appendicectomies performed after the introduction of our diagnostic pathway also decreased, which implies that there are unnecessary operations being avoided.

In the paediatric population, the use of CT is more difficult to justify compared to other imaging modalities. One major drawback of the CT scan is its high radiation exposure, which can increase the risk of cancer, especially in younger patients. The estimated lifetime attributable risk of cancer from a single abdominal-pelvic CT scan is reported to be 0.1% to 0.5% in young patients, and higher in younger females [14]. Therefore, clinical diagnosis or the use of other imaging modalities is generally preferred in paediatric or pregnant patients.

The use of MRI in the diagnosis of appendicitis has been studied, showing both high sensitivity and specificity, similar to CT scans, without exposing patients to ionising radiation [15]. However, MRI scans typically take longer to perform than CT scans, which may limit the use of MRI in emergency settings due to the delay in care and potential morbidity related to this. Moreover, MRI may require more specialised equipment and expertise and may be less widely available than CT or ultrasound in some healthcare settings.

Ultrasound is a non-invasive imaging modality that has been widely used to diagnose appendicitis. Several studies have investigated the accuracy of ultrasound in the diagnosis of appendicitis, with pooled studies suggesting a sensitivity of 77% and specificity of 60%, although there is considerable variation among studies [16,17]. This variance leads to one of the main drawbacks of ultrasound, i.e., there is significant reliance on the operator of ultrasound versus the standardisation of CT [18]. Furthermore, our study showed modest sensitivity and poor specificity when examining the use of ultrasound.

Appendicectomy is the most common operation in the field of general surgery [19]. Therefore, a reduction in the NAR should decrease the number of hospital admissions. This is especially important for the individual patient, as no operation is without risk. Apart from the risks related to general anaesthesia, operations carry morbidity risks in terms of pain, wound infection, and formation of adhesions, which seem hard to justify in view of a healthy appendix being removed [20].

Furthermore, when comparing the price of a single CT scan to an unnecessary operation, the increased use of preoperative imaging could save costs for individual hospitals, allowing for better allocation of resources. Appendicectomies are often done in the emergency theatre, and reducing the burden from negative appendicectomies would allow better use of this scarce resource.

There are, however, several limitations to our study. It was performed at a single centre, and this work would ideally have to be conducted at multiple sites to validate our findings. Furthermore, the post-intervention portion of this study was performed during the height of the COVID-19 pandemic, and the increased use of preoperative CT may be explained by the increased need of ensuring that operating was only done when absolutely necessary, rather than being affected by the introduction of our diagnostic pathway. The data collection period could be expanded in the future at a time when the pandemic has subsided to assess whether our findings can be replicated.

## Conclusions

As the use of preoperative imaging has accelerated throughout the 21st century, the acceptance of a high NAR appears to be reducing over time. The morbidity related to a potentially unnecessary operation should be avoided whenever possible. The use of preoperative CT, in particular, appears to provide the greatest benefit in reducing the NAR. In the older population, the radiation risk is less amplified, and the use of imaging in this patient population is easy to justify. However, in children and younger adults, particularly females of childbearing age, the potential radiation risk may be more difficult to justify. For these subsets of patients, there appear to be several options. Ultrasound does not carry radiation risk but appears to be less reliable when compared to CT. The use of MRI appears to offer the same diagnostic accuracy as CT but is not as ubiquitous as CT. Finally, the option to accept the NAR in this population of patients is an option. In conclusion, preoperative imaging does reduce the NAR, but this effect is only established in patients who undergo preoperative CT, and there are limitations to patients who can undergo this imaging modality.

## References

[1] Nguyen A, Lotfollahzadeh S (2023). Appendectomy. https://pubmed.ncbi.nlm.nih.gov/35593822/.

[2] Snyder MJ, Guthrie M, Cagle S (2018). Acute appendicitis: efficient diagnosis and management. Am Fam Physician.

[3] Noureldin K, Hatim Ali AA, Issa M, Shah H, Ayantunde B, Ayantunde A (2022). Negative appendicectomy rate: incidence and predictors. Cureus.

[4] Chaochankit W, Boocha A, Samphao S (2022). Negative appendectomy rate in patients diagnosed with acute appendicitis. BMC Surg.

[5] Jeon BG (2017). Predictive factors and outcomes of negative appendectomy. Am J Surg.

[6] Ferris M, Quan S, Kaplan BS (2017). The global incidence of appendicitis: a systematic review of population-based studies. Ann Surg.

[7] Wickramasinghe DP, Xavier C, Samarasekera DN (2021). The worldwide epidemiology of acute appendicitis: an analysis of the global health data exchange dataset. World J Surg.

[8] Bhangu A, Søreide K, Di Saverio S, Assarsson JH, Drake FT (2015). Acute appendicitis: modern understanding of pathogenesis, diagnosis, and management. Lancet.

[9] Petroianu A (2012). Diagnosis of acute appendicitis. Int J Surg.

[10] Hansson J, Körner U, Ludwigs K, Johnsson E, Jönsson C, Lundholm K (2012). Antibiotics as first-line therapy for acute appendicitis: evidence for a change in clinical practice. World J Surg.

[11] Raja AS, Wright C, Sodickson AD (2010). Negative appendectomy rate in the era of CT: an 18-year perspective. Radiology.

[12] Maghrebi H, Maghraoui H, Makni A (2018). [Role of the Alvarado score in the diagnosis of acute appendicitis]. Pan Afr Med J.

[13] Chan J, Fan KS, Mak TL, Loh SY, Ng SW, Adapala R (2020). Pre-operative imaging can reduce negative appendectomy rate in acute appendicitis. Ulster Med J.

[14] Miglioretti DL, Johnson E, Williams A (2013). The use of computed tomography in pediatrics and the associated radiation exposure and estimated cancer risk. JAMA Pediatr.

[15] D'Souza N, Hicks G, Beable R, Higginson A, Rud B (2021). Magnetic resonance imaging (MRI) for diagnosis of acute appendicitis. Cochrane Database Syst Rev.

[16] Fu J, Zhou X, Chen L, Lu S (2021). Abdominal ultrasound and its diagnostic accuracy in diagnosing acute appendicitis: a meta-analysis. Front Surg.

[17] Lehmann B, Koeferli U, Sauter TC, Exadaktylos A, Hautz WE (2022). Diagnostic accuracy of a pragmatic, ultrasound-based approach to adult patients with suspected acute appendicitis in the ED. Emerg Med J.

[18] Corson-Knowles D, Russell FM (2018). Clinical ultrasound is safe and highly specific for acute appendicitis in moderate to high pre-test probability patients. West J Emerg Med.

[19] Ramsay G, Wohlgemut JM, Jansen JO (2018). Emergency general surgery in the United Kingdom: a lot of general, not many emergencies, and not much surgery. J Trauma Acute Care Surg.

[20] Calis H (2018). Morbidity and mortality in appendicitis in the elderly. J Coll Physicians Surg Pak.

